# Central nervous system tumors: a single center pathology review of 34,140 cases over 60 years

**DOI:** 10.1186/1472-6890-13-14

**Published:** 2013-05-02

**Authors:** Liang Chen, Xiang Zou, Yin Wang, Ying Mao, Liangfu Zhou

**Affiliations:** 1 Department of Neurosurgery, Huashan Hospital, Fudan University, 12 Wu Lu Mu Qi Zhong Road, Shanghai, Jing An District, 200040, China; 2 Department of Neuropathology, Huashan Hospital, Fudan University, 12 Wu Lu Mu Qi Zhong Road, Shanghai, Jing An District, 200040, China

**Keywords:** Central nervous system tumors, Epidemiology, Pathological review, Single center, WHO 2007 classification

## Abstract

**Background:**

Tumor epidemiology is a significant part of CNS (central nervous system) tumor studies. Reassessment of original sections can update our knowledge of tumor spectrum. Here, we discuss the features of CNS tumor pathology in a single center.

**Methods:**

A total of 34140 cases from 1950 to 2009 were collected; sections from 1990 to 2009 were reassessed according to WHO 2007 classification, and cases from 1950 to 1989 were classified according to the previous pathological diagnosis.

**Results:**

Seven CNS tumor categories during 1990 to 2009 were as follow: neuroepithelial tissue (38.0%), tumors of the meninges (36.5%), tumors of the sellar region (4.1%), germ cell tumors (1.3%), tumors of cranial and paraspinal nerves (13.3%), lymphomas and hematopoietic neoplasm (1.7%), metastatic tumors (5.1%), where histological types by age and sex were diverse. Overall, males exceeded females in distributions of most CNS tumor subtypes, while tumors of the meninges occurred more frequently in females. The case number of lymphomas and hematopoietic neoplasms grew the fastest during the past five years, and the distribution of neuroepithelial tumors remained stable over the past twenty years.

**Conclusions:**

Despite the possibilities of cross sample biases, the data in this series could suggest a similar CNS tumor spectrum as might occur in other developing countries.

## Background

Central nervous system (CNS) tumors are not as frequent as tumors of many other sites, such as those of the reparative or digestive systems [1], but their incidence rate has increased over time. A report from the International Agency for Research on Cancer (IARC) revealed that the worldwide incidence rate of CNS tumors in 2002, which was age-adjusted and considered the standard world population, was 3.7/100,000 persons among males and 2.6/100,000 persons among females. The incidence rates were higher in developed countries (males: 5.8/100,000 persons; females: 4.1/100,000 persons) than in less developed countries (males: 3.0/100,000 persons; females: 2.1/100,000 persons) [2]. In 2008, the rates had risen to 3.8/100,000 persons in males and 3.1/100,000 persons in females, although the incidence rates in developed countries (males: 5.8/100,000 persons; females: 4.4/100,000 persons) still remained higher than those in less developed countries (males: 3.2/100,000 persons; females: 2.8/100,000 persons). An overall increase has been observed throughout the world, especially in less developed countries, which has captured our attention.

The Neurosurgical Department in Huashan Hospital is a main clinical center for neurological diseases in China. In this study, we retrospectively retrieved the neuropathological data obtained from our hospital during the period of 1950-2009 and analyzed the spatiotemporal changes relative to the published data over the past six decades from other countries.

## Methods

This study was performed at the Neurosurgical Department of the Huashan Hospital, Fudan University. Ethical approval was granted by the ethics committee of Huashan Hospital. The cases in this report were collected from patients who were diagnosed with CNS tumors and who were surgically treated or received biopsies between 1950 and 2009. Due to the loss of the original pathological sections, the cases from 1950 to 1989 could not be reassessed directly. In those cases, crude classifications were performed according to the previous pathological diagnoses that were used and adopted by neurosurgeons and neuropathologists at the time. However, our cases from 1990 to 2009 were reassessed by neuropathologists from the Neuropathology Department of Huashan Hospital according to the standards from the 2007 World Health Organization (WHO) classification [3].

All the CNS tumors were divided into seven categories: tumors of neuroepithelial tissue; tumors of the cranial and paraspinal nerves; tumors of the meninges; lymphomas and hematopoietic neoplasms; germ cell tumors; tumors of the sellar region; and metastatic tumors. Some differences exist between the WHO 2007 and WHO 2000 classifications; for example, gliomatosis cerebri was newly included in astrocytic tumors, and pilomyxoid astrocytoma was added as a new subtype in pilocytic astrocytoma. Consequently, we reassessed all the pilocytic astrocytoma cases to separate the cases of pilomyxoid astrocytoma from the old histological diagnoses. Extraventricular neurocytoma, papillary glioneuronal tumor, and rosette-forming glioneuronal tumor of the fourth ventricle were added to a neuronal and mixed neuronal-glial tumors subtype. Angiocentric glioma was grouped with other neuroepithelial tumors. For choroid plexus, atypical choroid plexus papilloma was inserted between choroid plexus papilloma and choroid plexus carcinoma [4].

The WHO classification offers a crude histological grading system, in which each CNS tumor is classified as grade I-IV according to its degree of malignancy. This system can provide an estimate for the prognosis of a patient. In this study, age, sex, and the tumor histological type and grade were systematically recorded. In the detailed classified cases (1990-2009), the gender data were lost for 83 of the patients, and the age data were lost for 180 of the patients, representing 0.30% and 0.65% of the total, respectively. Consequently, we excluded the lost gender or age cases in the statistics of the sample. All the data analyses were performed using SPSS (Statistical Package for the Social Sciences) software.

## Results

### General features

From 1950 to 1989, 6338 cases could be grouped according to their CNS tumors based on our crude classification. Among these cases, 3530 were male, 2734 were female, and 935 were children and teenagers (age 0-19). Further details are provided in Additional files 1 and 2.

From 1990 to 2009, 27,802 out of 43,595 cases with brain disease could be grouped into CNS tumors according to the 2007 WHO classification system. The other diseases included pituitary adenoma for 7843 cases, cavernous malformation for 1247 cases, arteriovenous malformation for 848 cases, and other CNS diseases for 5854 cases (epidermoid cysts, parasites, abscesses, and others). The proportion of CNS tumors was 63.8%. Among these cases, 13945 were males, 13774 were females, and 2903 were children and teenagers (age 0-19). The distribution of the CNS tumors by histological subtypes is shown in Table 1. Of the malignant tumors, glioblastoma was the most common, with a proportion of 29.5%. Metastatic tumor was the next common and represented 19.4% of the total. These tumor types were followed by anaplastic astrocytomas, anaplastic oligodendromas, and others. The details are presented in Table 1. A few rare CNS tumors were encountered in this series, including new entities in the 2007 WHO classification, such as anaplastic hemangiopericytoma (8 cases), papillary tumors of the pineal region (2 cases), atypical choroid plexus papillomas (2 cases), rosette-forming glioneuronal tumor of the fourth ventricle (4 cases), and papillary glioneuronal tumors (13 cases).

**Table 1:** Histological features of 27802 cases from 1990 to 2009

	**N (%)**	**M**	**F**	**Ma(yrs)**	**Ma(yrs)**
I. Tumors of neuroepithelial tissue					
1. Astrocytic tumors					
Pilocytic astrocytoma	469 (1.69)	253	214	21.1	17
Pilomyxoid astrocytoma	20 (0.07)	8	12	19.2	17
Subependymal giant cell astrocytoma	27 (0.10)	15	12	16.2	13
Pleomorphic xanthoastrocytoma	67 (0.24)	33	34	28.4	23
Glioblastoma	2170 (7.81)	1362	805	50.2	53
Giant cell glioblastoma	12 (0.04)	9	3	42.7	41
Gliosarcoma	64 (0.23)	39	25	48.8	50.5
Anaplastic astrocytoma	939 (3.38)	593	346	49.2	42
Gliomatosis cerebri	3 (0.01)	2	1	59	62
2. Oligodendroglial tumors					
Oligodendroglioma	990 (3.56)	555	426	38.6	39
Anaplastic oligodendrogliom	534 (1.92)	313	221	43.5	44
3. Oligoastrocytic tumors					
Oligoastrocytoma	243 (0.87)	143	100	38.9	39
Anaplastic oligoastrocytoma	89 (0.32)	56	33	44.7	46
4. Ependymal tumors					
Ependymoma	623 (2.24)	350	273	37	38
Subependymoma	39 (0.14)	21	18	43	43
Anaplastic ependymoma	223 (0.80)	129	93	30	30
5. Choroid plexus tumor					
Choroid plexus papilloma	91 (0.33)	34	57	29	29
Atypical choroid plexus papilloma	2 (0.01)	0	2	37.5	37.5
Choroid plexus carcinoma	11 (0.04)	8	3	31.6	25
6. Other neuroepithelial tumors					
Chordoid glioma of the third ventricle	3 (0.01)	1	2	31.3	27
Astroblastoma	4 (0.01)	2	2	37.8	42
7. Neuronal and mixed neuronal-glial tumors					
Cerebellar liponeurocytoma	1 (0.00)	1	0	59	59
Desmoplastic infantile astrocytoma/ganglioglioma	5 (0.02)	1	4	16.8	11
Dysembryoplastic neuroepithelial tumor	54 (0.19)	34	20	22.6	17.5
Dysplastic gangliocytoma of cerebellum, Lhermitte-Duclos	7 (0.03)	3	4	28.7	38
Rosette-forming glioneuronal tumor of the fourth ventricle	4 (0.01)	0	4	28.3	24
Paraganglioma	29 (0.10)	14	15	42.5	42
Papillary glioneuronal tumor	13 (0.05)	9	4	32.4	29
Anaplastic ganglioglioma	43 (0.15)	26	17	39.7	41
Gangliocytoma	40 (0.14)	29	11	29.6	31.5
Ganglioglioma	137 (0.49)	87	49	33.1	32
Central neurocytoma	131 (0.47)	63	68	30.2	29
8. Tumors of pineal region					
Papillary tumor of the pineal region	2 (0.01)	1	1	31.5	31.5
Pineocytoma	15 (0.05)	5	10	30.3	22
Pineal parenchymal tumor of intermediate differentiation	5 (0.02)	5	0	26.8	20
Pineoblastoma	10 (0.04)	5	5	25.6	17.5
9. Embryonal tumors					
Medulloblastoma	515 (1.85)	329	183	15.4	12
PNET	119 (0.43)	75	43	21.9	17
Atypical teratoid/rhabdoid tumor	4 (0.01)	2	2	21.8	23
II. Tumors of cranial and paraspinal nerves					
1. Schwannoma (neurilemoma, neurinoma)	3499 (12.59)	1754	1736	45	45
2. Neurofibroma	166 (0.60)	106	60	36.3	38
3. Perineurioma	1 (0.00)	0	1	69	69
4. MPNST	26 (0.09)	11	15	37.8	42
III. Tumors of meninges					
1. Tumors of meningothelial cells					
Grade I meningothelial cells tumors	7623 (27.42)	2294	5309	50.7	51
Atypical	359 (1.29)	184	173	51.2	52
Chordoid	16 (0.06)	8	8	48.5	51
Clear cell	30 (0.11)	13	17	37.5	39.5
Papillary	26 (0.09)	16	10	36.7	35.5
Rhabdoid	15 (0.05)	9	6	42.2	42
Anaplastic, malignant	235 (0.85)	129	105	50.3	50
2. Mesenchymal tumors					
Hemangiopericytoma	202 (0.73)	112	88	43.3	42
Anaplstic hemangiopericytoma	8 (0.03)	5	3	48.3	45.5
Other Mesenchymal tumors	825 (2.97)	450	369	36	37
3. Primarymelanocytic lesions					
Diffuse melanocytosis	2 (0.01)	1	1	35.5	35.5
Melanocytoma	9 (0.03)	5	4	47	47
Malignantmelanoma	31 (0.11)	21	10	42.2	42
4. Other neoplasms related to the meninges					
Hemangioblastoma	760 (2.73)	463	293	40.8	40
IV. Lymphomas and hematopoietic neoplasms	464 (1.67)	272	191	49.7	52
V. Germ cell tumors					
1. Germinoma	204 (0.73)	148	56	17.4	16
2. Embryonal carcinoma	14 (0.05)	12	2	12.2	12.5
3. Yolk sac tumor	2 (0.01)	2	0	7	7
4. Choriocarcinoma	1 (0.00)	0	1	34	34
5. Teratoma	10 (0.04)	8	2	14.5	14
Mature	56 (0.20)	36	20	23.5	20.5
Immature	22 (0.08)	20	2	16	14.5
With malignant transformation	4 (0.01)	4	0	13.3	14
6. Mixed germ cell tumors	35 (0.13)	29	6	16.6	14
VI. Tumors of the sellar region					
Craniopharyngioma	1144 (4.11)	665	477	32.1	33
Granular cell tumor	7 (0.03)	2	5	49	52
Pituicytoma	1 (0.00)	1	0	45	45
Spindle cell oncocytoma of the adenohypophysis	1 (0.00)	1	0	24	24
VII. Metastatic tumors	1431 (5.15)	890	536	53.9	55
Total	27802	13945	13774		

***N*** = Number; ***M*** = Male; ***F*** = Female;

***Ma*** = Median age at diagnosis; ***ma*** = Mean age at diagnosis;

***PNET*** = Primitive neuroectodermal tumor;

**Grade I meningothelial cells tumors** = Meningiothelial; Fibrous, fibroblastic; Transitional, mixed; Psammomatous; Angiomatous; Microcystic; Secretory; Lymphop lasmacyte-rich; Metaplastic.

**Other mesenchymal tumors** = Lipoma; Angiolipoma; Hibernoma; Liposarcoma; Solitary fibrous tumor; Fibrosarcoma; Malignant fibrous histiocytoma; Leiomyoma; Leiomyosarcoma; Rhabdomyoma; Rhabdomyosarcoma; Chondroma; Chondrosarcoma; Osteoma; Osteosarcoma; Osteochondroma; Hemangioma; Angiosarcoma; Kaposi sarcoma; Ewing sarcoma-PNET.

***MPNST*** = Malignant peripheral nerve sheath tumor.

### Histological types by age and sex

Gender differences in morbidity were common in many of the subtypes. From 1962 to 1989, all the CNS tumor subtypes (except tumors of the meninges) occurred more frequently in males (Additional file 1). Additionally, from 1990 to 2009, neuroepithelial tumors (the most common category) were observed more frequently in male patients, with a ratio of 1.47 according to this survey. Tumors of the meninges exhibited a female/male ratio that exceeded 1.7 according to our data (Table 1), whereas males were more susceptible to germ cell tumors, with a ratio of 2.91.

**Figure 1:**
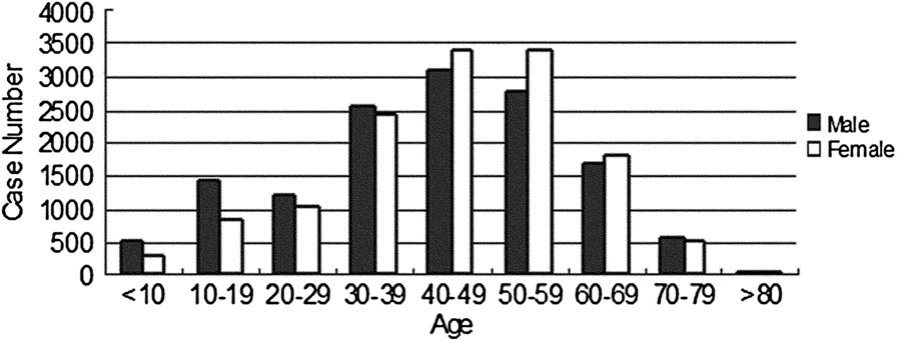
Distribution of CNS tumors by age and sex from 1990 to 2009.

The tumor distribution by age is another impressive aspect of brain tumor epidemiology. The histogram presented in Figure 1 shows a peak proportion within the age range from 30 to 60 in males, whereas the female patients seemed to peak at an older age relative to the males. There was another proportional peak from 10 to 19 years of age in the male patients, and malignant tumors tended to occur more frequently at that age. In adults, the top three subtypes included tumors of the meningothelial cells (33.6%), schwannoma (14.2%), and astrocytic tumors (11.4%). For children and teenagers (age 0-19), astrocytomas were the most common CNS tumors (29.2%), followed by medulloblastoma (13.1%) and craniopharyngioma (12.3%) (Table 2).

**Table 2:** Distributions of CNS tumors and top 4 subtypes among children and teenagers (age 0-19)

**Tumor**	**N**	**M**	**F**	**%**
Tumors of neuroepithelial tissue	1791	1086	700	61.3
Astrocytoma	853	489	361	29.2
Medulloblastoma	383	254	128	13.1
Ependymal tumours	180	113	67	6.2
Tumors of the sellar region	378	231	157	12.9
Craniopharyngioma	358	224	134	12.3
Tumors of the meninges	326	204	120	11.2
Germ cell tumors	237	182	54	8.1
Tumors of cranial and paraspinal nerves	163	94	69	5.6
Lympnomas and hematopoietic neoplasms	21	16	5	0.7
Metastatic tumors	6	3	3	0.2
Total	2922	1816	1108	100

***N*** = Number; ***M*** = Male; ***F*** = Female.

**Figure 2:**
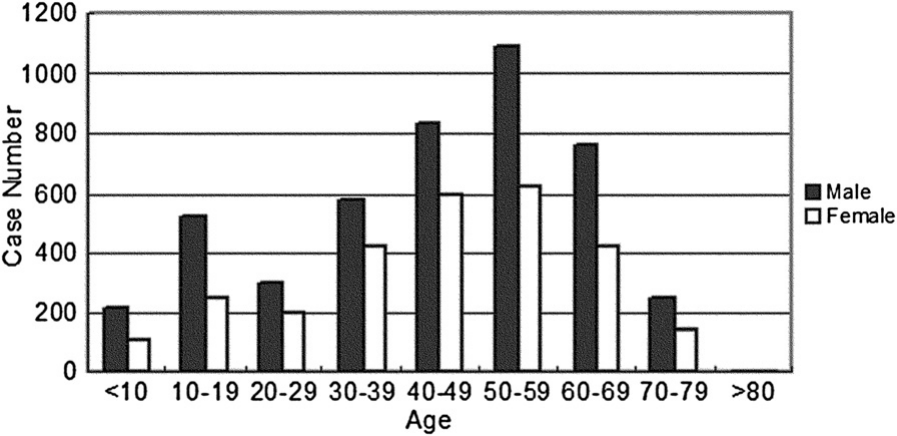
Distribution of malignant tumors (WHO III-IV) by age and sex from 1990 to 2009.

**Figure 3:**
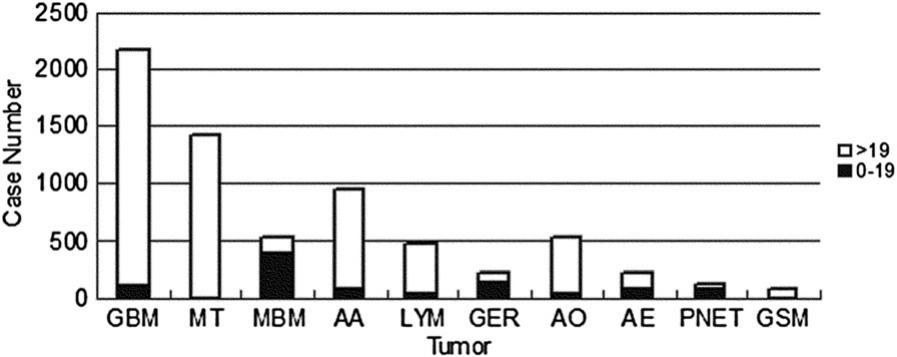
**Some malignant tumors in children and elders from 1990 to 2009.** GBM = Glioblastoma; MT = Metastatic Tumor; MBM = Medulloblastoma; AA = Anaplastic astrocytoma; LYM = Lymphoma; GER = Germinoma; AO = Anaplastic oligodendrocytoma; AE = Anaplastic ependymoma; PNET = Primitive neuroectodermal tumor; GSM = Gliosarcoma.

For the WHO III-IV grade tumors, two incidence peaks were identified in both male and female patients, with malignant tumors occurring more often in males at each age group (Figure 2). The spectra of the malignant tumors were different in the pediatric and adult groups (Figure 3). For the adults, glioblastoma, metastatic tumor, and anaplastic astrocytoma occupied the top three places. However, for the pediatric cases (age 0-19), the top three malignant tumors included medulloblastoma, germ cell tumors, and glioblastoma.

### Time trends over the 60-year period

**Figure 4:**
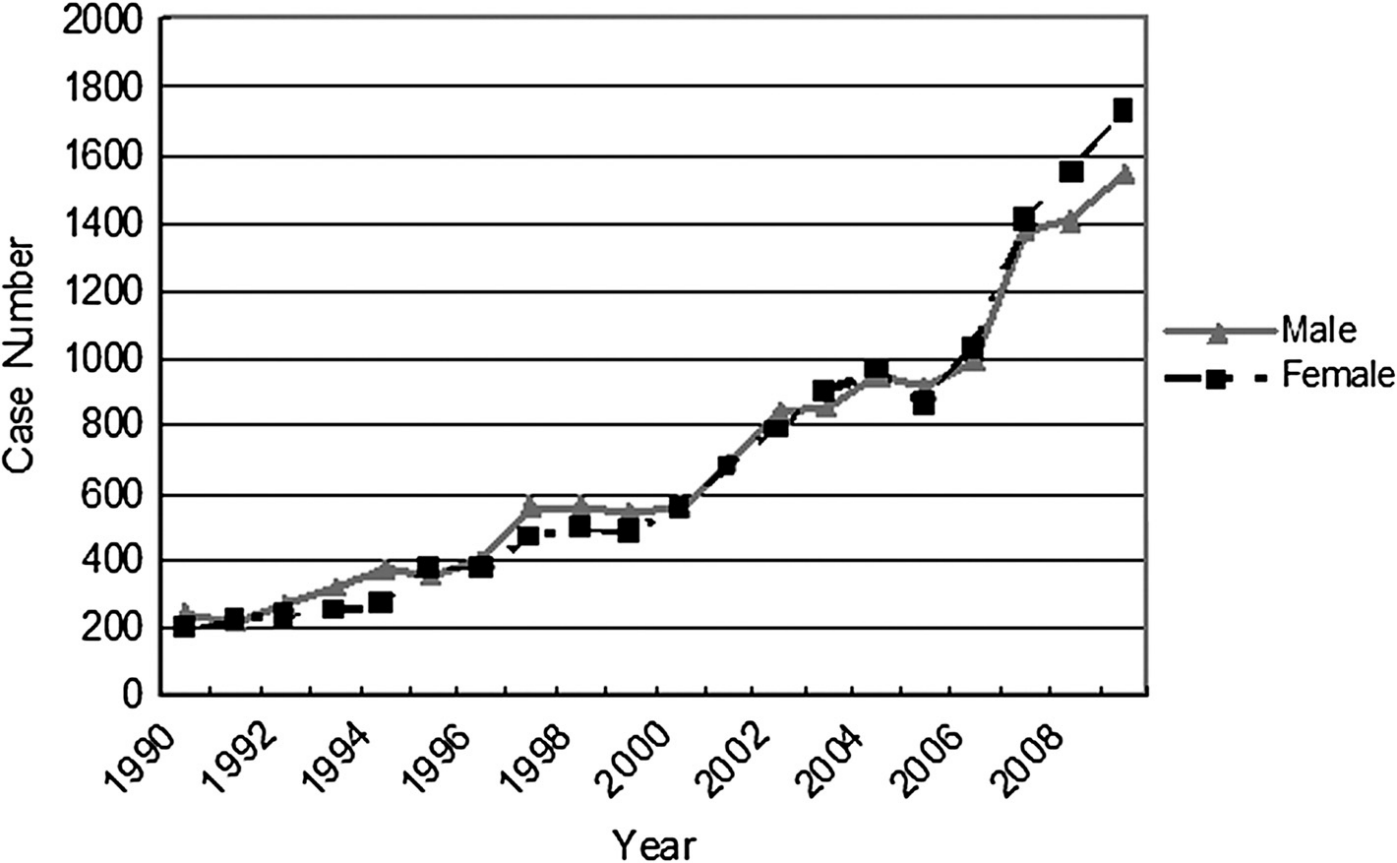
Time trends from 1990 to 2009 by sex.

Over the past six decades, the number of patients has grown tremendously. In contrast to 72 cases in 1962, there were 3275 cases in 2009 (Additional file 3 & Figure 4), and the growth rate had reached 106% to 139% in the three most recent years. This trend was primarily the result of a continuous enlargement of the hospital and the neurosurgical department, with other possible reasons that will be discussed below. Gender differences were also noticeable. From 1962 to 2006, male patients seemed to be the susceptible population of CNS tumors, whereas over the last three years, the situation reversed. The female/male ratios were 1.02, 1.10, and 1.12 from 2007 to 2009, showing an increasing trend.

**Figure 5:**
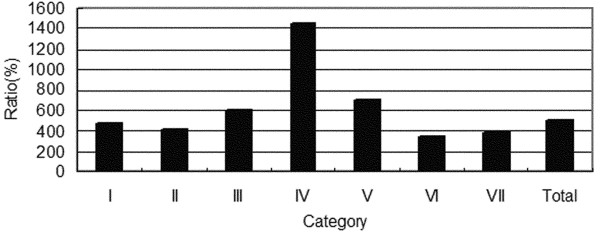
**Growth rate of CNS tumors (90-94/05-09).** I = Tumors of neuroepithelial tissue; II = Tumors of cranial and paraspinal nerves; III = Tumors of meninges; IV = Lymphomas and hematopoietic neoplasms; V = Germ cell tumors; VI = Tumors of the sellar region; VII = Metastatic tumors.

**Figure 6:**
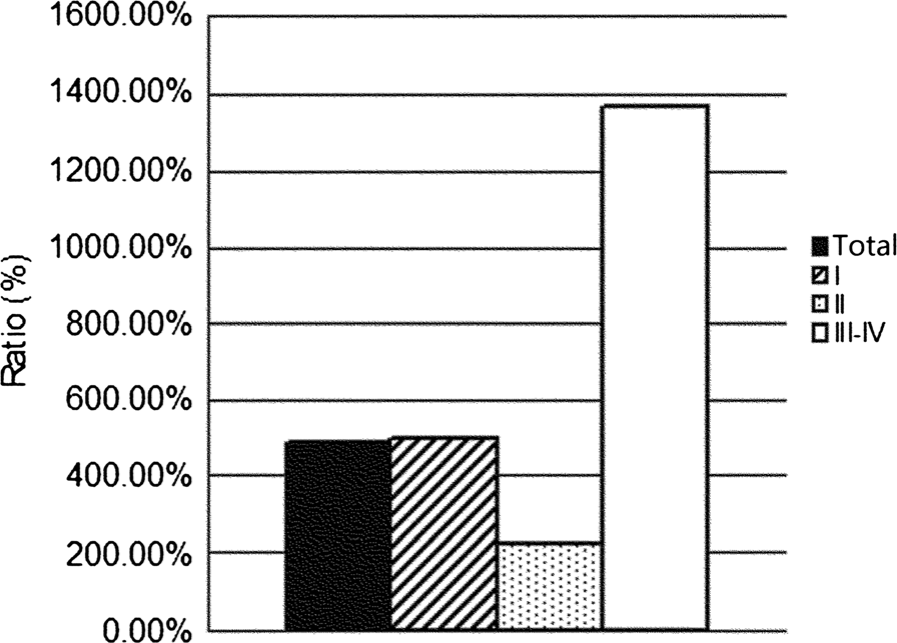
Growth Rate (05-09/90-94) of CNS tumors by WHO grade.

**Figure 7:**
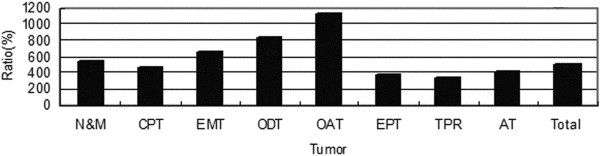
**Growth rate of neuroepithelial tumors.** N&M = Neuronal and mixed neuronal-glial tumors; CPT = Choroid plexus tumors; EMT = Embryonal tumors; ODT = Oligodendroglial tumors; OAT = Oligoastrocytic tumors; EPT = Ependymal tumors; TPR = Tumors of pineal region; AT = Astrocytic tumors.

**Figure 8:**
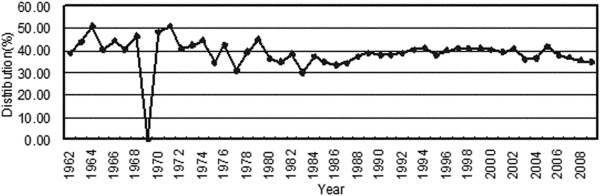
Distribution of neuroepithelial tumors by time.

Although the incidence rates of all the CNS tumors increased rapidly, certain subtypes of these tumors exhibited remarkable increases (Figure 5). Lymphomas and hematopoietic neoplasms were 14.6-fold more common during 2005-2009 than during 1990-1994, whereas the incidence of all CNS tumors increased only 4.9-fold. The second highest increase in incidence was found for germ cell tumors (7-fold), followed by meningiomas (6-fold). The incidence of grade III-IV tumors increased much more rapidly than that of grade I-II tumors and the average incidence rate (Figure 6). The mean increase in neuroepithelial tumors was equal to the average increase, but diverse incidence rates existed among the subtypes. Oligodendrogliomas and oligoastrocytomas increased respectively 1.7 and 2.3 times faster than average (Figure 7). However, the distribution of neuroepithelial tumors, the most common category of CNS tumors, remained stable throughout the 20-year period (Figure 8).

## Discussion

A retrospective epidemiological review of brain tumors is particularly important for future research because it can demonstrate the changes in the tumor spectrum of a population, reveal possible risk factors, and indicating potential therapy methods. China is the most populous country in the world, but it has not yet established a national incidence rate for CNS tumors. Over the past 20 years, acknowledged improvements have occurred in the economy and medical services in China, with concurrent environmental and lifestyle changes. Therefore, the epidemiological characteristics of China can be helpful for a worldwide study and may help predict possible future changes in undeveloped areas as they undergo development. Finally, because of the differences that are present in each classification edition, the reassessment of past cases is required to update our knowledge of CNS tumors.

The distribution of CNS tumor categories found in this study did not exactly replicate the rates observed in previously published worldwide reports. Despite the selection bias inherent in this single-center study, tumors of neuroepithelial tissue and the meninges were the two most common categories; the proportions were 38.0% and 36.5%, respectively. A nationwide database in France revealed that the proportions of these two categories were 53.9% and 28.8%, respectively, from 2004-2008 [5], whereas another population-based report from CBTRUS (Central Brain Tumor Registry of the United States) recorded rates of 33.7% and 35.5%, respectively, from 2004-2007 [6]. The reason for this great variation remains unknown and requires further investigation.

The tumor spectra varied from adults to children and teenagers as well as from males to females. According to the CBTRUS data, glioblastoma (17.7%) and anaplastic astrocytoma (2.1%) were the most common malignant tumors in adults. In this study population, metastatic tumor, glioblastoma, and anaplastic astrocytoma a larger proportion in adults, whereas medulloblastoma, germ cell tumors, and glioblastoma were more common among children and teenagers.

Among the pediatric cases, brain and spinal tumors have been shown to be the second most common cancers (20.2%) in Shanghai, China, based on a previous regional study [7]. High proportions of astrocytomas (29.2%) and medulloblastomas (12.3%) were found in children and teenagers in our series, which seems to be in accordance with reports from other locations, such as Taiwan (population-based study), France (population-based study), India (single-center study), and Brazil (single-center study) [8-11]. However, a population-based study in Japan reported a lower proportion of medulloblastomas relative to germ cell tumors and craniopharyngiomas [12], whereas in France (8.6%) and India (5.2%), ependymal tumors were observed at higher proportions relative to germ cell tumors [8,9]. Details are provided in Table 3. During the past 20 years, children under the age of 4 were not permitted admission to our center; therefore, the spectrum of pediatric CNS tumors in our series might be distorted. The epidemiological variability in the observed histopathology worldwide indicates that the origin of these tumors might be closely related to racial and environmental factors.

**Table 3:** Proportions of top CNS tumor subtypes among children and teenagers in different regions

				**Astrocytoma**	**Medulloblastoma**	**Craniopharyngioma**	**Germ cell tumors**	**Ependymal tumours**
**Country**	**Author**	**Period**	**Age(yrs)**					
China		1990-2009	0-19	29.2%	13.1%	12.3%	8.1%	6.2%
Taipei	Wong et al. [11]	1975-2004	0-18	36.9%	15.8%	9.8	12.7%	6.8%
Japan	Makino et al. [12]	1989-2008	0-15	38.6%	13.1%	13.7	14.4%	5.2%
India	Asirvatham et al. [8]	1990-2004	0-18	49.7%	12.0%	10.3%	3.0%	5.2%
France	Bauchet et al. [9]	2004-2006	0-19	32.9%	13.1%	5.4%	3.6%	8.6%
Brazil	Pinho et al. [10]	1989-2009	0-21	37.7%	16.3%	12.5%	7.0%	8.1%
US	CBTRUS [6]	2004-2007	0-19	33.1%	12.0%	3.6%	4.8%	6.7%

Overall, a larger proportion of males had malignant CNS tumors across all the age groups in this limited population. Data from CBTRUS also revealed a male/female incidence ratio of 1.38 during 2003-2007. For the neuroepithelial tumors, the ratio (male/female) was 1.47 compared to 1.25 (2004-2007) from CBTRUS. Therefore, according to this survey and to other regional studies [13-18], it can be assumed that males tend to have a higher incidence of CNS tumors. Because of the high incidence of neuroepithelial tumors in men, gender difference could be a starting research point that may lead to some valuable findings with respect to tumor origins and chemotherapy. Gender difference found for meninges tumors in our survey was also striking, although we found a ratio (female/male) of only 1.7 compared to the 2.68 ratio based on the CBTRUS data (2004-2007). However, due to the widespread use of advanced diagnostic technologies in China, we predict that this ratio will increase in the near future.

For children and teenagers, the male/female ratio for CNS tumors was 1.66 in the selected cases. Other studies around the world have reported similar ratios: 1.1 in France (population-based study), 1.31 in Japan (population-based study), and 1.27 in Brazil (single-center study) [9,10,12]. From our data, males accounted for 74.4% of the germ cell tumors in the pediatric cases, a percentage that was similar to the 69.6% reported by CBTRUS and to those reported by other studies from various regions [8-12], indicating that intrinsic factors also play important roles in oncogenesis.

Several studies have also revealed an increasing trend in the incidence rate for CNS tumors in western countries. In the United States, a population-based series showed that the annual percent change (APC) was +1.1% from 1985-1999 [19], whereas in France, an APC of +2.33% was observed from 2000 to 2007 [13]. Clinically silent CNS tumors have been identified in a larger proportion of the population, particularly among the elderly, because of improved imaging techniques [17,20]. Furthermore, the increasing trend was more prominent among certain CNS tumor subtypes [17,21-23]. xThe increased incidences of gliomas and meningiomas were suspected to have resulted from the widespread use of cell phones since the mid-1990s, but no causal association was confirmed [24,25]. A larger sample size, a uniform method of classification, and a longer observation time are necessary before we can reach a definitive conclusion.

In China, a fast-growing and developing country, increasing tumor morbidity has been blamed as one of the top three causes of death [1], just as in most western countries. Because it was not a population-based study, the APC in this series reached +11.22%. In part, this increase could be the result of the increase in hospital size, with the increased number of cases due to more sickrooms and operating rooms, improved neurosurgical techniques making more cases operable and treatable, and economic developments that result in more affordable therapy for most patients. Another key reason might be associated with an increased detection rate of CNS tumors after the widespread use of CT and MRI in both rural and urban areas in China, which reinforced the referral bias. In the early 1980s, the first CT machine was imported into Huashan hospital; this was also the first CT machine in China. In the late 1980s, the first MRI followed. Those machines contributed greatly to the diagnosis of CNS tumors, and from Additional file 3, we indeed observe a continuous increase in the number of cases during that period. Moreover, because the textile industry was booming in a developing China during the 1980s, women constituted the majority of textile workers. Therefore, occupation-related risk factors were simultaneously raised, and a case–cohort study showed a higher brain tumor incidence in female textile workers during that period in Shanghai [26]. The agreement with this data series suggests that the large and detailed database from our sample is very valuable because it reveals how quickly the CNS tumor spectrum changed in China with respect to the affected population characteristics.

The diverse growth rates of the CNS tumor subtypes are also very worthy of consideration. The fastest growing categories of tumors in this study included lymphomas, germ cell tumors, and tumors of the meninges. Lymphoma has been ranked as one of the top ten malignant tumors in this century, and it has the fastest growing incidence rate of all malignant tumors in China [27]. Despite the inherent biases, certain chemical pollutants should be carefully considered [28,29]. Germ cell tumors are also thought to be closely related to environmental factors. They exhibit a considerable geographical variation in incidence, representing 3-15% of all primary pediatric intracranial neoplasms [30]. Reports from Japan revealed a step-up increase in the incidence from 1980 to 1993 and indicated that winter-born children were more susceptible [31,32]. For the tumors of the meninges, increasing trends were observed around the world [13,17,19,21-23,31], and improvements in the diagnostic technologies are still considered to be the major causal factor. Worldwide studies have also demonstrated an apparently increased incidence of metastatic tumors [33], but this increase was less prominent in our series. The difference might be an artifact of this investigation, as this is a pathological-based review; thus, brain metastasis could have been detected earlier and have been well controlled by radiosurgery without pathological proof. According to our study, most of the fastest increasing subtypes were also malignant, which underscored our need to be more cautious. Therefore, we need more large-scale population-based studies to identify the real risk factors.

As a single-center retrospective series, the data in this study could not represent the national epidemiology of CNS tumors. The pathological diagnoses included in this study were only from patients under surgery or biopsy. Furthermore, surgical treatments were not commonly used in some advanced malignant tumors. Therefore, the distribution of some of the tumor subtypes might be artificially high or low due to the unclear denominator. In brief, various biases were unavoidable in this study, which means that the conclusions should be evaluated with caution.

## Conclusions

This study retrospectively analyzed a large-scale and long-term pathological database from a single neurological center. Although it was not a large population study, and a selection bias inevitably existed, the rapidity of the observed changes in the CNS tumor spectrum with respect to the affected population characteristics in China could model similar changes that will occur in other developing countries.

## Competing interests

The authors declare that they have no competing interests.

## Authors’ contributions

LZ: He has made substantial contributions to conception and design, and acquisition of funding. LC and XZ: They had made contributions equally to acquisition of data, analysis and interpretation of data, and drafting the manuscript. YW and YM: They had made contributions to revising the manuscript critically, and had given final approval of the version to be published. All authors read and approved the final manuscript.

## Pre-publication history

The pre-publication history for this paper can be accessed here:

http://www.biomedcentral.com/1472-6890/13/14/prepub

## Supplementary Material

Additional file 1 Distribution of CNS Tumor Categories by Sex from 1962 to 1989. Click here for file

Additional file 2 **Distribution of CNS Tumor Categories by Age from 1962 to 1989.** I = Tumors of neuroepithelial tissue; II = Tumors of cranial and paraspinal nerves; III = Tumors of meninges; IV = Lymphomas and hematopoietic neoplasms; V = Germ cell tumors; VI = Tumors of the sellar region; VII = Metastatic tumors. Click here for file

Additional file 3 Time Trends from 1962 to 1989 by Sex. Click here for file
